# Influence on Soybean Aphid by the Tripartite Interaction between Soybean, a Rhizobium Bacterium, and an Arbuscular Mycorrhizal Fungus

**DOI:** 10.3390/microorganisms10061196

**Published:** 2022-06-11

**Authors:** Élisée Emmanuel Dabré, Mohamed Hijri, Colin Favret

**Affiliations:** 1 Institut de Recherche en Biologie Végétale, Département de Sciences Biologiques, Université de Montréal, 4101 rue Sherbrooke Est, Montréal, QC H1X 2B2, Canada; mohamed.hijri@umontreal.ca (M.H.); colinfavret@aphidnet.org (C.F.); 2 African Genome Center, Mohammed VI Polytechnic University (UM6P), Lot 660, Hay Moulay Rachid, Ben Guerir 43150, Morocco

**Keywords:** arbuscular mycorrhizal fungi, *Aphis glycines*, *Bradyrhizobium japonicum*, co-inoculation, microorganism–plant–insect interactions, *Rhizophagus irregularis*, symbiosis

## Abstract

The inoculation of arbuscular mycorrhizal (AM) fungi and rhizobia in legumes has been proven to increase plant growth and yield. To date, studies of the effects of these interactions on phytophagous insects have shown them to be context-dependent depending on the inoculant strain, the plant, and the insect species. Here, we document how a symbiosis involving an AM fungus, *Rhizophagus irregularis*; a rhizobium, *Bradyrhizobium japonicum*; and soybean, *Glycine max*, influences the soybean aphid, *Aphis glycines*. Soybean co-inoculated with the AM fungus–rhizobium pair increased the plant’s biomass, nodulation, mycorrhizal colonization, nitrogen, and carbon concentrations, but decreased phosphorus concentration. Similar effects were observed with rhizobium alone, with the exception that root biomass was unaffected. With AM fungus alone, we only observed an increase in mycorrhizal colonization and phosphorus concentration. The aphids experienced an increased reproductive rate with the double inoculation, followed by rhizobium alone, whereas no effect was observed with the AM fungus. The size of individual aphids was not affected. Furthermore, we found positive correlation between nitrogen concentration and aphid population density. Our results confirm that co-inoculation of two symbionts can enhance both plant and phytophagous insect performance beyond what either symbiont can contribute alone.

## 1. Introduction

The soybean aphid, *Aphis glycines* Matsumura (Hemiptera: Aphididae), is an invasive pest of cultivated soybean, *Glycine max* (L.) Merr. (Fabaceae), in North America [1]. Native to Asia, it was discovered in North America in 2000 and quickly became established in at least 30 U.S. states and 3 Canadian provinces [2,3]. Soybean aphid has a high reproductive capacity, with populations doubling under favorable conditions in as few as 1.5 to 2 days [4]. It is a specialist phloem-feeder and is an important invasive pest of soybean, its secondary host [5]. Aphid feeding can reduce plant height, pod set, and seed size; decrease seed oil content; but increase protein content [6,7]. Yield reductions of up to 40% occur under heavy aphid pressure [8].

Environmental factors such as nutrient availability affect a plant’s susceptibility to herbivory [9], and nutrient limitation to the plant can in turn affect the nutritional value of the plant to herbivores [10]. One of the most important dietary factors influencing the performance of herbivorous insects is the nitrogen concentration in their host: piercing–sucking insects, such as aphids, show a particularly strong response to nitrogen level in their host plants [11]. The growth and reproduction of aphids are affected by the accessibility of essential nutrients in the phloem sap of host plants [12].

Like most aphids, the limiting component of the soybean aphid diet is often nitrogen, which is in relatively low concentrations within host phloem [13,14]. Studies have linked the population growth rate of the soybean aphid to nitrogen availability in soybeans [15]: population growth increases when the aphids are on plants with increased phloem nitrogen concentration, such as on plants growing in fields with potassium-deficient soils [12,14,16,17], or on plant growth stages where nitrogen is more readily available.

Soybean is the most important agricultural legume for oil and protein production [18]. This crop is typically used in rotation with corn, a system that dominates the landscape in the North-Central United States of America (USA) and Ontario and Quebec in Canada. One of the ecological factors that can change soybean plant traits is root-associated beneficial organisms such as arbuscular mycorrhizal (AM) fungi and bacterial rhizobia, the most important microbes associated with the plant [19]. Arbuscular mycorrhizal fungi ubiquitously form symbiotic associations with the roots of most terrestrial plants [20]. In most cases, AM fungi improve plant growth and the nutritional status for phytophagous insects by increasing the acquisition of soil nutrients such as phosphate [21]. At the same time, rhizobia are bacteria that are associated with most legumes with which they form root nodules and enhance nitrogen status through biological fixation of dinitrogen N_2_ [22]. Therefore, soybean can form tripartite symbiotic associations with AM fungi and nodule-inducing rhizobia simultaneously, which may increase both P and N, respectively, in the plant [23].

Meeting the increasing global demand for food requires efficient use of finite resources and presents a key challenge for agriculture [24]. In this context, agricultural sustainability is gaining importance and has seen the development and use of commercial biological inoculants (bacteria and/or fungi) to increase the mobilization of key nutrients, especially phosphorus (P) and nitrogen (N), and enhance their availability to crop plants [25,26,27]. Microbial inoculants also help mitigate environmental impacts caused by agrochemicals [28]. Nowadays, the use of microbial inoculants in agriculture is spread worldwide for different crops and involves different microorganisms [29]. In particular, soybean is currently the most inoculant-consuming crop worldwide, associating with bacteria belonging to the genus *Bradyrhizobium* [28].

The increased plant growth and improved nutrient uptake resulting from the use of these organisms as inoculants could make plants more attractive or sensitive to herbivores [30]. Like other phloem-feeding insects, soybean aphid is generally affected positively by AM fungi [31], but in most studies conducted in controlled conditions, the effects of AM fungi-induced changes on this insect vary from positive to negative [32]. Similarly, the symbiosis of rhizobia with legumes that influences plant–herbivore interactions is also context-dependent [33]. It is known that the presence of rhizobia and AM fungi leads to increased plant nutrient uptake [27,34], which presumably can affect insect herbivores such as aphids.

In this study, we examined the effects of a tripartite association involving the AM fungus *Rhizophagus irregularis* (Błaszk., Wubet, Renker, and Buscot) C. Walker and A. Schüßler, the rhizobium *Bradyrhizobium japonicum* (Kirchner) Jordan, and soybean *Glycine max* cv. ETNA, on the health of *Aphis glycines* in controlled circumstances. We hypothesized that the inoculation of either microbial symbiont alone, or the co-inoculation of both together, would increase the reproductive rate of soybean aphid on its host. Secondly, we expected that the symbionts would increase individual aphid growth, resulting in larger adults. Thirdly, we predicted that the expected increase in the rate of aphid reproduction would be correlated with a change in nutrient concentration in the plant brought about by the symbionts.

## 2. Materials and Methods

### 2.1. Plant Material, Bacteria and Fungi Strains, and Insect Samples

In this study, seeds of the soybean cultivar ETNA and commercial inoculants were supplied by Premier Tech (Rivière-du-Loup, QC, Canada). The mycorrhizal inoculant contained 500 spores per gram of *R. irregularis* isolate DAOM197198, the most prevalent commercially available AM fungi for the last two decades [26]. The rhizobial inoculant was a liquid solution containing *B. japonicum* PTB 162 at a dose of 7 × 10^9^ colony-forming units per 1 mL of inoculum.

We used lab-reared individuals from the entomological lab of Jacques Brodeur (Plant Biology Research Institute (IRBV), University of Montreal, Montreal, QC, Canada). Aphid colonies were maintained on soybean, *G. max*. cv. ETNA, until used for the experiments. Slide-mounted voucher specimens are deposited in the University of Montreal Ouellet-Robert Entomological Collection, catalog numbers QMOR61147–QMOR61152.

### 2.2. Growth Conditions, Inoculation, and Insect Treatment

The experiment consisted of a randomized block design with and without mycorrhizal and rhizobial inoculants. Blocks of four treatments were replicated eight times, for a total of 32 experimental plots.

Soybean seeds were sterilized following the protocol described by Premier Tech (unpublished data, see Supplementary Materials Protocol S1). Seeds were sterilized in 95% ethanol for 30 s, rinsed with water several times, and then treated with 3% peroxide solution for 10 min and rinsed with water several times. They were germinated on 1.5% agar (Sigma, Lisboa, Portugal) for three days before sowing. Sowing was carried out in 45–50 mL germinating cells with potting substrate PRO-MIX PGX containing vermiculite (Premier Tech). For each treatment of AM fungus, 2 g of commercial inoculum containing 1000 spores was added [35]. For the bacterial treatment, 40 µL of inoculum was used for each treatment (Premier Tech). All plants were grown in a walk-in growth chamber CMP 3244 (Conviron, Manitoba, Canada) at 20 °C (day) and 18 °C (night), L16:D8 photoperiod, and at 55–65% relative humidity for the duration of the experiment. Two weeks later, the plants were transferred into three-liter pots (6.5″ × 7″) on sterilized potting substrate composed of BM6 soil, a horticultural coarse sphagnum peat mix (80–90%), and perlite (Terris, QC, Canada). Plants were fertilized with 50 mL of standard nutrient Long Ashton solution [36] once per week and watered twice weekly with the same quantity of water.

Six weeks later, the plants had attained the third trifoliate stage. We used two clip-cages held in place by metal rods, one on a leaf of the second trifoliate and a second on a leaf of the third trifoliate [37]. Four apterous adult aphids of *A. glycines* from the same source colony on soybean were introduced into each clip-cage on the underside of the leaf and left to reproduce parthenogenetically for 48 h. These four adults were removed, and the number of aphid nymphs was standardized at five per clip-cage. One observation was made each day for over three weeks as the colonies grew. The soybean aphid takes approximately 6.9 days to complete its cycle at 20 °C [4,38]; we thus observed them for approximately three generations. After this time had passed, the number of aphids (abundance) was recorded a final time, and the body and tibia lengths of all adult aphids preserved in 70% ethanol were measured using a Zeiss V20 stereomicroscope and Zeiss Zen Blue imaging software (Zeiss, Oberkochen, Germany).

### 2.3. Analysis of Arbuscular Mycorrhizal Colonization and Nodule Counting 

The plants were analyzed six weeks after transplanting. To estimate the mycorrhizal colonization rate, we randomly sampled 30 separate 1 cm root sections from each plant, for a total of 960 root sections. The root samples were boiled for 1 h in 10% (wt/vol) KOH solution at 90 °C. Later, the roots were placed in 5% acetic acid for 4 min before staining with a solution of 0.5% trypan blue for 40 min at 90 °C. Roots were then de-stained with distilled water for 20 min and observed under a compound microscope at 100× magnification. To confirm that the inoculations worked, the percentage of the root colonized was determined by the gridline intersect method [39,40], and the number of nodules was counted (see Table 1).

### 2.4. Analysis of Fresh and Dry Biomass and Nitrogen, Phosphorus, and Carbon Content and Concentration

Fresh weight was measured for the shoot and root components of each plant. These were then oven-dried at 65 °C for 72 h and reweighed to obtain their dry mass. Nitrogen and carbon concentrations of shoots were determined by combustion with an Elementar total C/N analyzer, Thermo Fisons, model EA1108 (Chemistry Laboratory, University of Montreal). Phosphorus concentration was measured using a UV spectrophotometer, Shimadzu UV-1800, at a wavelength of 712 nm, in the digested shoot solution following the method of Murphy and Riley (1962) [41] adapted by the Pedology Laboratory (University of Montreal).

### 2.5. Statistical Analysis

Analyses were performed with R [42]. To examine the effects of AMF and rhizobia inoculation on plant parameters (shoot and root fresh and dry weight biomass; nitrogen, phosphorus, and carbon concentrations; nodulation; and AM fungus colonization rate) and the number of aphids, we used the function lmer in the package lme4 [43] to perform linear mixed-effects models. Inoculant treatments were used as a fixed factor and blocks were used as a random factor. The normality of the distribution and the homogeneity of the variance were tested by the Shapiro–Wilk and Levene’s tests, respectively. When the model was established, we applied the function of analysis of variance in the package “car” to test the significance. In cases when a significant difference was observed, a post hoc test was applied with Tukey’s honest significant difference (HSD) in the package “multcomp”. As the response variable “number of aphids” did not meet the assumption of normality and homogeneity, we applied the function sqrt (square root) to transform the variable [44]. The variables aphid size and tibia length were fitted with the function aov to perform linear model analysis by ANOVA. To assess the influence of the inoculation on aphids, we evaluated possible correlations between insect parameters (final abundance, aphid size, tibia length) and plant biomass, nutrient concentration, and content (dry biomass, nitrogen, phosphorus, carbon) by performing Kendall correlation analyses [45].

## 3. Results

### 3.1. Effects of Inoculation on Plant Parameters

The experiment consisted of a randomized block design with four levels of microbial inoculant, namely control (M−R−), mycorrhizal inoculant (M+R−), rhizobium inoculant (M−R+), and double inoculant mycorrhiza and rhizobium (M+R+). The double inoculant (M+R+) had a higher level of mycorrhizal colonization compared to AM fungus alone (M+R−). Likewise, root nodulation was highest with the double inoculant (M+R+) and the rhizobium treatment (M−R+), whereas there was no difference between them.

The shoot fresh mass and dry mass were significantly different between treatments. The plants with the double inoculant (M+R+) had the highest above-ground (shoot) mass, followed by the rhizobium treatment (M−R+); no difference was observed between the control (M−R−) and mycorrhizal (M+R−) treatments (Table 1). The double inoculant (M+R+) also yielded the highest root fresh and dry mass, relative to the control (M−R−); however, the root mass of the plants in the rhizobium (M−R+) and mycorrhizal (M+R−) treatments was intermediate and did not show any statistical difference (Table 1).

We measured the plant’s total nitrogen, phosphorus, and carbon content. As these elements influence plant growth, and as aphids should be more sensitive to plant nutritional quality than to host size, we also calculated their respective concentrations relative to plant dry biomass. Overall plant nitrogen content was higher with the double inoculant (M+R+) followed by rhizobium inoculant (M−R+) than the control (M−R−) and AM fungi inoculants (M+R−) which were not different from each other (Table 1). Nitrogen concentration (content controlled for plant mass) was also higher with the double inoculant (M+R+) and rhizobium inoculant (M−R+), which were similar relative to the control (M−R−) and AM fungus inoculant (M+R−). Phosphorus content was highest with AM fungi-inoculated plants ((M+R+) and (M+R−)) while phosphorus concentration was highest with the AM fungus inoculant (M+R−), followed by the control (M−R−) and rhizobium (M−R+); the plants with the double inoculant (M+R+) had the lowest P concentration. The double-inoculated plants (M+R+) had the higher carbon content, followed by rhizobium-inoculated plants (M−R+), as compared to the control (M−R−) and the AM fungi-inoculated plants (M+R−). The carbon concentrations of rhizobia-inoculated plants ((M+R+) and (M−R+)) were similar and higher relative to the AM fungi-inoculated (M+R−) and control plants (M−R−), which were not different from each other (Table 1). The ratio N:P followed the same trend as nitrogen content where the double inoculant (M+R+) had the highest value, followed by rhizobium inoculant (M−R+), compared to the control (M−R−) and AM fungus inoculant (M+R−) (Table 1). With the P:N, the control (M−R−) and AM fungus (M+R−) treatments had the highest values compared to the other inoculants.

### 3.2. Effects of Inoculation on Soybean Aphid

**Figure 1:**
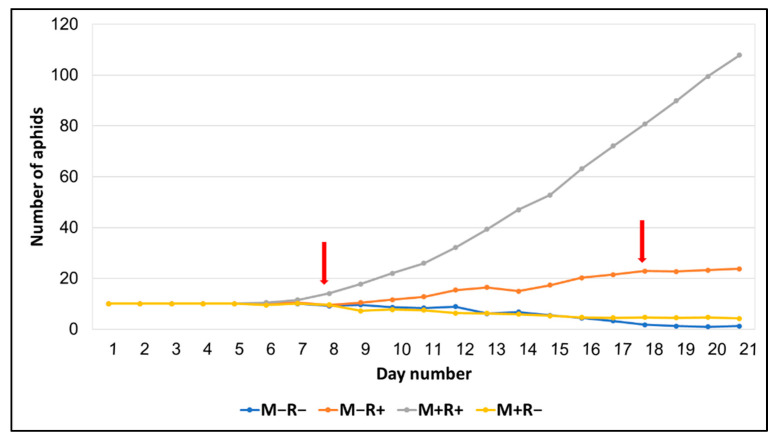
Aphid colony growth over three weeks as a function of inoculant treatment (control (M−R−); mycorrhizae and rhizobium (M+R+); mycorrhizae alone (M+R−); rhizobium alone (M−R+)). The arrows indicate the days at which the aphid populations were first divergent.

After standardizing the number of first instar aphids per plant, we measured the reproductive rate of aphids. Figure 1 shows the daily evolution of aphid colony growth over three weeks. A difference between the effects of inoculants on aphid population density was first observed on day number 8 (first red arrow in Figure 1, Table 2). The double inoculant (M+R+) had the highest number of aphids relative to the other inoculant treatments, which were all similar until day number 18 (Table 2). On that day, three inoculant treatments were measurably different from each other (second red arrow in Figure 1). After 3 weeks of observation, the double treatment (M+R+) still showed the highest number of aphids, followed by the rhizobium treatment (M−R+) (Table 2). Aphid colonies failed to grow on control (M−R−) and mycorrhizal treatments (M+R−) (Figure 1). We also measured the body length and left hind tibia length of 20 aphids per treatment. No differences were observed between the four treatments (Table 2).

### 3.3. Correlation between Aphid Abundance and Plant Parameters

Plant nutritional content was significantly correlated with the aphid population, nodulation, AM fungus colonization, and shoot and root mass. Nitrogen, phosphorus, and carbon content were positively correlated with all these variables (Supplementary Materials Table S1). In addition, plant nutritional concentration was significantly correlated with the abundance of aphids. Nitrogen concentration was positively correlated with aphid colony size such that when the nitrogen concentration was higher, there was an increase in aphid colony size (Table 3). Contrary to the trend with nitrogen, an increase in phosphorus concentration (as opposed to content) was correlated with a decrease in aphid colony size. We also observed a significant correlation between aphid body size and aphid left hind tibia length, but neither was related to treatment (Supplementary Materials Table S1).

## 4. Discussion

This study demonstrated that the tripartite symbiotic association between AM fungus, rhizobium, and soybean alters the parameters of the plant such as shoot and root mass; nitrogen, phosphorus, and carbon concentrations and contents; root mycorrhizal colonization; and nodulation (Table 1). The mycorrhization and nodulation observed on inoculated-plant roots are evidence of the establishment of symbiosis between AM fungus/rhizobium and soybean that led to changes in nutritional status and shoot mass (Table 1). The increased phosphorus concentration and content measured with AM fungus-inoculated plants are in line with the studies of [35,44]. Furthermore, some studies similarly found an increase in nitrogen and carbon concentrations and shoot mass in plants associated with nitrogen-fixing bacteria [46,47]. Our study also confirmed the co-inoculation effects of AM fungi and rhizobia with an increase in N and P content in inoculated plants, as documented by [48].

The nutritional quality of a plant influences its suitability as a food resource for herbivores. In our study, changes in inoculated plants increased the aphid colony growth and hence the eventual abundance of the soybean aphid, but they did not affect the size of the individual aphids (Table 1 and Table 2). Rhizobium nodulation increased plant nitrogen concentration and decreased phosphorus concentration, while at the same time positively affecting aphid colony growth. No such relation was observed with the AM fungus colonization (Table 2 and Table 3). Unlike mycorrhizal fungi, showing generally positive effects on specialist sap-feeders [31], the effects of rhizobia on insect herbivores appear to be context-dependent [33]. Brunner et al. (2015) [49] found that soybean plants infected with *Bradyrhizobium japonicum* had reduced soybean aphid densities. Similarly, there was a significant reduction in aphid colony size when *B. japonicum* was associated with *Delftia acidovorans* (den Dooren de Jong) Wen et al., a plant growth promoter producing the phytohormone indole-3-acetic acid (IAA) [50], or with lipochitooligosaccharides or Nod factors, molecules involved in symbiosis [49,51]. Previously, Dean et al. (2009) found that soybean plants associated with introduced *B. japonicum* did not affect soybean aphid densities relative to naturally occurring rhizobial strains which in fact reduced aphid densities [46]. They also found that introduced strains of *B. japonicum* were genetically different from natural strains. Finally, they did not link the increase in aphid population by indigenous strains of *B. japonicum* with plant parameters (size, leaf concentrations of N and P, and nodulation), which were similar among rhizobia-associated treatments. The same authors in another study showed no effects of soybean root nodulation by *B. japonicum* on soybean aphid population growth [52]. However, our study showed an increase in the number of aphids with the rhizobia-inoculated plants, with a positive relation between nitrogen uptake and nodulation.

In addition, we found positive correlations between nodulation and aphid densities, nodulation and nitrogen concentration, and nitrogen concentration and aphid colony growth (Table 3). Given the nitrogen-limited diet of phloem feeders [12,53,54], it is unsurprising that nodulation, and hence nitrogen content, and hence aphid performance would all be positively correlated.

The effects of symbiotic interactions between AM fungi and plants on insects are well documented [35,55,56,57,58,59]. AM fungus-induced changes in the plant are known to affect the performance of phytophagous insects, but such effects may be variable depending on the strain of the AM fungus, the associated plant, and the insect species [32,57,60]. As mentioned above, AM fungus seems to positively affect the abundance of phloem-feeders [31,35,61], as a consequence of P uptake in plants. However, we found no effect of AM fungus on the population growth of soybean aphids despite the increase in shoot phosphorus concentration (Table 1). On the contrary, we found a negative correlation between P concentration and aphid colony growth. In a previous study, Babikova et al. (2014) showed an increase in pea aphids (*Acyrthosiphon pisum* (Harris)) on AM fungi-infected broad bean plants (*Vicea faba* L.) [62]. However, there was no effect of mycorrhizal colonization on P and N leaf concentration, and this did not explain the growth of the aphid population. Some studies also show that the population growth rate and development time of phytophagous insects are influenced not only by plant nutrient levels but also by nutrient ratios [63]. In our study, N outweighed phosphorus in the ratio N:P and positively influenced the aphid population; whereas in the ratio P:N, it was phosphorus that negatively affected aphid colony growth.

The association between AM fungus and rhizobia (i.e., the M+R+ treatment) increased the population of aphids, followed by the rhizobium inoculant alone (i.e., M−R+), relative to the other inoculant treatments (Table 2). Some studies have documented a synergistic effect between AM fungus and rhizobia on soybean growth and an increase in plant N and P content relative to each inoculant alone [48,64]. For example, co-inoculation of soybean roots with *Bradyrhizobium japonicum* or *Bradyrhizobium* sp. considerably enhanced root colonization by the mycorrhizal fungus *Glomus mosseae* (T.H. Nicolson and Gerd.) Gerd. and Trappe (syn. *Funneliformis mosseae*) and increased N and P uptake by the plant [48,65]. In our study, we found the same result with symbiont association and nutrient uptake but opposite effects for N and P concentrations: the former increased whereas the latter decreased. Rhizobium alone or associated with AM fungus increased nitrogen concentration, but decreased phosphorus concentration. A hypothesis that could explain this situation is that the rhizobium plays a key role in the increase in N ((M−R+) versus (M+R+) inoculants) but inhibits the uptake of P by AM fungus ((M+R−) and (M+R+) inoculants) (Table 1). In contrast, both N and P content were highest in plants co-inoculated with AM fungus–rhizobium than in those inoculated with either alone (Table 1). For example, despite a low P and the same N content relative to other inoculants, the double inoculation resulted in the highest aphid population growth. Another explanation is that there is a relation between nutrient contents and concentration with a positive relation for nitrogen but no clear relation with phosphorus. As both parameters were correlated with shoot dry mass, positively in the case of nitrogen and negatively in that of phosphorus, we expect shoot dry mass to influence phosphorus status in the plant and its involvement in aphid nutrition and growth. In the case of this association between rhizobia and AM fungi, nitrogen concentration is not the sole factor contributing to the increased population growth of soybean aphids, as shown with the case of phosphorus in plants associated with AM fungi [44].

In conclusion, our results documented a complex and synergistic interaction between inoculation with rhizobia and AM fungi and soybean growth and downstream effects on soybean aphid performance. The outcomes of this study are that inoculation of individual AM fungus and rhizobium showed an increase in P and N contents and concentrations, respectively. With co-inoculation, we found a higher N content and similar N concentration compared to rhizobium alone. There was similar P content with AM fungus–rhizobium inoculant and a decrease in P concentration relative to AM fungus alone, as well as an increased plant biomass compared to either inoculant alone. In addition, we confirmed that when the AM fungus and rhizobium are co-inoculated, they produced a synergistic effect that is more beneficial to the aphids than either inoculant alone. In an agricultural context, AM fungi and rhizobia inoculants used as biological fertilizers may help crop yield by enhancing plant growth and nutrient content, but they may also have deleterious effects by favoring insect pests.

Finally, it is not clear why the AM fungus inoculant did not stimulate aphid colony growth despite an increase both in P concentration and in mycorrhizal colonization of roots. Indeed, the negative correlation between P and aphid abundance suggests that other factors remain to be elucidated, perhaps involving the resistance mechanisms of the plant. As beneficial organisms, symbiotic microbes can change host plant traits for insect herbivores through their impact on plant nutritional quality and/or by priming effects that lead to enhanced inducible and constitutive plant defenses [33,66]. We suggest further study on soybean aphid focusing simultaneously on the direct and indirect effects of inoculation.

## Figures and Tables

**Table 1:** Plant parameters (mean ± SE, F, P, df = 3) of 8 replicates per inoculant treatment. Control, M−R−; mycorrhizae + rhizobium, M+R+; mycorrhizae, M+R−; rhizobium, M−R+. Linear mixed-effects model (LMM) followed by ANOVA indicates differences among treatments. Letters following the mean ± SE represent Tukey’s honest significant difference (HSD) groupings.

Plant Parameters	Inoculant Treatments				*F*	*p*
	M−R−	M+R−	M−R+	M+R+		
AM fungus root colonization (%)	0	21.7 ± 3.05	0	37.4 ± 6.31	32.32	<0.0001
Number of nodules	5.12 ± 2.20 a	3.50 ± 1.32 a	44.38 ± 7.21 b	56.50 ± 3.25 b	44.54	<0.0001
Shoot fresh biomass (g/plant)	4.15 ± 0.60 a	6.81 ± 0.72 ab	10.03 ± 0.92 b	17.93 ± 0.30 c	41.73	<0.0001
Shoot dry biomass (g/plant)	1.05 ± 0.10 a	1.49 ± 0.16 ab	2.40 ± 0.23 b	4.64 ± 0.40 c	40.94	<0.0001
Root fresh biomass (g/plant)	4.66 ± 0.80 a	5.78 ± 0.47 ab	5.70 ± 0.49 ab	7.85 ± 0.71 b	4.38	0.012
Root dry biomass (g/plant)	0.31 ± 0.05 a	0.41 ± 0.04 ab	0.43 ± 0.03 ab	0.60 ± 0.06 b	5.91	0.003
Total nitrogen (mg/plant)	22.3 ± 4.41 a	43.9 ± 9.13 a	97.4 ± 11.13 b	176.9 ± 11.69 c	59.40	<0.0001
Total phosphorus (mg/plant)	5.95 ± 0.80 a	9.58 ± 0.87 b	7.99 ± 0.40 ab	9.97 ± 0.77 b	7.18	0.0016
Total carbon (mg/plant)	464 ± 45.90 a	649 ± 74.03 ab	1070 ± 105.40 b	2053 ± 170.34 c	43.06	<0.0001
Nitrogen concentration (mg/g)	20.1 ± 2.9 a	27.0 ± 2.8 a	39.4 ± 1.0 b	38.2 ± 1.4 b	27.27	<0.0001
Phosphorus concentration (mg/g)	5.25 ± 0.52 c	6.45 ± 0.31 d	3.46 ± 0.30 b	2.22 ± 0.22 a	34.78	<0.0001
Carbon concentration (mg/g)	431 ± 3.0 ab	426 ± 3.0 a	438 ± 2.6 b	438 ± 3.9 b	5.75	0.005
Ratio N:P	4.30 ± 0.91 a	4.35 ± 0.54 a	12.13 ± 1.30 b	18.28 ± 1.61 c	38.80	<0.0001
Ratio P:N	3.00 ± 0.53 b	2.62 ± 0.36 b	0.89 ± 0.09 a	0.58 ± 0.05 a	36.93	<0.0001

**Table 2:** The average number of aphids, individual size (body length), and tibia length (mean ± SE, F, P, df = 3) of 8 replicates per treatment (control (M−R−); AM fungus and rhizobium (M+R+); AM fungus alone (M+R−); rhizobium alone (M−R+)). Linear mixed-effects model (LMM) for aphid number and linear model for aphid body and tibia length, followed by ANOVA, indicates differences among treatments. Letters followed by mean ± SE represent Tukey’s honest significant difference (HSD) groupings. Different letters indicate significant differences between inoculant treatments, whereas the same letter indicates no difference. sqrt: *square root*.

Aphid Parameters	Inoculant Treatments				*F*	*p*
	M−R−	M+R−	M−R+	M+R+		
Aphid number (8 days)	9.12 ± 0.40 a	9.50 ± 0.46 a	9.62 ± 0.46 a	14.12 ± 1.77 b	6.31	0.003
Aphid number (18 days)	1.75 ± 1.06 a	4.62 ± 2.96 a	22.88 ± 5.8 b	80.75 ± 15.6 c	41.71	0.0001
Aphid number (21 days) (sqrt)	0.60 ± 0.35 a	1.30 ± 0.60 a	4.57 ± 0.64 b	9.94 ± 1.13 c	16.47	0.001
Aphid body length (µm)	1304 ± 32 a	1421 ± 30 a	1323 ± 41 a	1320 ± 40 a	2.68	0.105
Left hind tibia length (µm)	661 ± 18.9 a	692 ± 18.1 a	667 ± 25.4 a	662 ± 22.4 a	0.70	0.403

**Table 3:** Correlation between plant and aphid variables. Nutrient concentrations are shown. For total nutrient content, see Supplementary Materials Table S1.

Variables A	Variables B	Kendall *tau* Coefficient	*p*
Aphid size	Tibia length	0.45	<0.0001
Final aphid colony size	Nitrogen concentration	0.57	<0.0001
	Phosphorus concentration	−0.58	<0.0001
	Carbon concentration	0.32	0.01
	Nodulation	0.63	<0.0001
	AM fungus colonization	0.35	0.009
	Shoot dry mass	0.74	<0.0001
	Root dry mass	0.48	0.0001
Nodulation	Nitrogen concentration	0.49	<0.0001
	Phosphorus concentration	−0.53	<0.0001
	Carbon concentration	0.27	0.03
	AM fungus colonization	0.18	0.18
	Shoot dry mass	0.64	0.0001
	Root dry mass	0.47	0.0002
AM fungus colonization	Nitrogen concentration	0.18	0.180
	Phosphorus concentration	−0.15	0.273
	Carbon concentration	0.03	0.821
	Shoot dry mass	0.39	0.003
	Root dry mass	0.36	0.007
Shoot dry mass	Nitrogen concentration	0.59	<0.0001
	Phosphorus concentration	−0.58	<0.0001
	Carbon concentration	0.33	0.008
Root dry mass	Nitrogen concentration	0.31	0.012
	Phosphorus concentration	−0.36	0.003
	Carbon concentration	0.22	0.077

## Data Availability

Not applicable.

## Supplementary Materials

The following supporting information can be downloaded at: https://www.mdpi.com/article/10.3390/microorganisms10061196/s1, Protocol S1: Steps of sterilization of soybean seeds that may contain *Bradyrhizobium* strains and other microorganisms (by Premier Tech). Table S1: Correlation between plant nutrient contents and aphid variables.
