# Supplementary material for: Influence on Soybean Aphid by the Tripartite Interaction between Soybean, a Rhizobium Bacterium, and an Arbuscular Mycorrhizal Fungus
Source: Microorganisms. 2022 Jun 11;10(6):1196. doi: 10.3390/microorganisms10061196 (PMC9228533; doi:10.3390/microorganisms10061196)
Supplement: Supplementary file 1 [file microorganisms-10-01196-s001.zip › microorganisms-1743655-supplementary.pdf]

Protocol S1: steps of sterilization of soybean seeds that may contain *Bradyrhizobium* strains and other microorganisms (by Premier Tech)

1. in a small Corpak-type container (250 ml), put the seeds and add 95% ethanol;

2. turn the container 1-2 times to coat the seeds (do not shake) and leave for 30 seconds;

3. Drain the seeds in a colander and rinse the Corpak container with sterile water;

4. return the seeds to the Corpak and add a 3% peroxide solution (10 volumes);

5. turn the Corpak over 1-2 times to wet the seeds and leave for 10 minutes;

6. Drain the seeds in a colander and rinse the Corpak with water;

7. Return the seeds to the Corpak and add sterile water and turn the container 1-2 times to rinse the seeds thoroughly (final rinse).

8. Sow the seeds immediately.

Table S1. Correlation between plant nutrient contents (nitrogen, phosphorus, carbon) and aphid colony size, and plants variables (nodulation, AM fungus root colonization, shoot and root dry mass).

**Variables A**	**Variables B**	**Kendall *tau* coefficient**	***p***
Final aphid colony size	Nitrogen content	0.77	<0.0001
	Phosphorus content	0.40	0.001
	Carbon content	0.76	<0.0001
Nodulation	Nitrogen content	0.68	<0.0001
	Phosphorus content	0.30	0.016
	Carbon content	0.64	<0.0001
AM fungus colonization	Nitrogen content	0.37	0.005
	Phosphorus content	0.42	0.001
	Carbon content	0.39	0.003
Shoot dry mass	Nitrogen content	0.91	<0.0001
	Phosphorus content	0.49	<0.0001
	Carbon content	0.99	<0.0001
Root dry mass	Nitrogen content	0.54	<0.0001
	Phosphorus content	0.40	0.0009
	Carbon content	0.56	<0.0001
